# Comparing Lifestyle Modifications and the Magnitude of Their Associated Benefit on Cancer Mortality

**DOI:** 10.3390/nu15092038

**Published:** 2023-04-23

**Authors:** Timothy P. Dougherty, Joshua E. Meyer

**Affiliations:** Department of Radiation Oncology, Fox Chase Cancer Center, Philadelphia, PA 19111-2497, USA

**Keywords:** dietary modification, cardiorespiratory fitness, physical activity, obesity, cancer mortality

## Abstract

Many cancers are associated with poor diet, lack of physical activity, and excess weight. Improving any of these three lifestyle factors would likely reduce cancer deaths. However, modifications to each of these—better nutrition, enhanced activity and fitness, and loss of extra body fat—have different effect sizes on cancer mortality. This review will highlight the relative benefit that each lifestyle change, enacted prior to a diagnosis of cancer, might impart on cancer-related deaths, as well as attempt to quantify the changes required to derive such a benefit. The review relies primarily on epidemiological data, with meta-analyses serving as the backbone for comparisons across interventions and individual studies within the larger meta-analyses providing the data necessary to form more quantitative conclusions. The reader can then use this information to better understand, recommend, and implement behaviors that might ultimately reduce cancer mortality. Of all the interventions, it seems clear that exercise, specifically improving cardiorespiratory fitness, is the best way to decrease the risk of dying from cancer.

## 1. Introduction

According to The World Cancer Fund, nearly one in five cancers in the United States is directly attributable to suboptimal diet, low physical activity, and/or excess adiposity [1,2]. This information clearly suggests that many cancers are preventable. However, the contribution of either nutrition or physical activity to risk reduction are quite varied in effect size. This review aims to highlight the different magnitudes that dietary modifications, physical activity, and excess adiposity might contribute to cancer mortality before a diagnosis of cancer.

Many of the studies cited in this article are epidemiological in nature. Many nutritional epidemiological studies rely on self-report, often food frequency questionnaires (FFQs), to record types of foods eaten (red meat, nuts, beverages, etc.). Although these FFQs have high reproducibility person to person and year to year [3,4], they do not necessarily relate well to total calorie intake and expenditure as suggested by doubly-labeled water experiments [5,6,7]. In addition, epidemiology does not establish causation, but rather association. To complicate matters, interpretation and application of epidemiological data, especially nutritional epidemiology, can be difficult [8]. Foods are also connected with one another, since a person needs to choose one meal or food group over another on a daily basis. Additionally, foods are often correlated—a person is more likely to consume a hamburger with a side of fries than a burger with a side of cashews. Lastly, differences between the highest and lowest intake of any food occupy a rather narrow range. For instance, the top and bottom quintiles of red meat intake are separated by about two-to-three servings, the equivalent of a half-pound steak. This may seem like a large difference, but compared to other variables, such as exercise, the room for variation is quite small.

Epidemiological studies involving exercise suffer slightly less from the aforementioned biases, primarily owing to the ability to directly measure baseline metrics, such as cardiorespiratory fitness (CRF) via exercise treadmill testing, or musculature, often via handgrip strength. These measures allow for more direct and quantitative metrics compared to FFQs. However, as with other epidemiological studies, large cohort studies looking at CRF and grip strength also suffer from healthy user bias and the lack of randomization. 

This review compiled the associated hazard ratio that several different lifestyle modifications have with respect to cancer mortality. In doing so, we attempted to offer a resource that allows the reader to compare the wide range of behavioral changes associated with reduced cancer-related deaths. We also attempted to contextualize the data with real-world examples that highlight the lifestyle habits necessary to potentially derive a cancer mortality benefit. Many of the articles cited in this review have a bias towards data collected in the United States. However, the content of this paper applies to any Westernized country or nation undergoing Westernization.

## 2. Methods

In order to compare the effect of various lifestyle modifications on cancer mortality, the hazard ratios from several different meta-analyses were amalgamated (Table 1). This served as our starting point. The respective meta-analyses were chosen via a PubMed search of the terms, “cancer mortality,” “meta-analysis,” and “[specific lifestyle behavior].” The “[specific lifestyle behavior]” regarding nutrition was searched as “[food group] consumption.” For instance, “nut consumption” was used to find meta-analyses related to nut intake and cancer mortality. The most highly cited meta-analysis written within the last 10 years was then chosen. If a search returned multiple meta-analyses, priority was given to the meta-analysis that employed the Preferred Reporting Items for Systematic Reviews and Meta-analysis Approach (PRISMA) [9] or Meta-analysis of Observational Studies in Epidemiology (MOOSE) [10,11] guidelines. Further preference was given to articles that used the Newcastle–Ottawa Scale to classify the quality of studies included within the respective meta-analysis. Lastly, papers that described the association between cancer mortality and high versus low groups were prioritized as to make comparisons across studies more understandable and to give context to the dose-relationships often reported in the literature. 

After finding an appropriate meta-analysis, the top two to three most highly weighted articles within the individual meta-analysis were then chosen to review. These individual papers were used to provide more quantitative insight into the differences between high and low (and occasionally middle/penultimate and low) quintiles (Table 2 and Table 3). In an attempt to contextualize some of these group differences, this paper interpreted some of the epidemiological data through the lens of the number needed to treat (NNT), which is the number of people required to undergo an intervention or dietary/lifestyle change in order to prevent one event, in this case, a death from cancer. The NNTs are based on raw data and so are uncorrected for confounders. Since population number and mortality rate can be found in individual studies, and not in meta-analyses, a singular study for each intervention was used to calculate the NNT. The study used to calculate the respective NNT was chosen if it had a similar hazard ratio (HR) to the one found in the meta-analysis on the particular subject. The HR, which gives the adjusted relative risk for cancer mortality, accompanies the NNTs for a more accurate, but sometimes conceptually more challenging, description of the data. Within the text, the NNT and HR are given for the population level (men and women combined), while Table 2 and Table 3 tabulate the NNTs and HRs for men and women, respectively. Lastly, Table 1 lists the adjusted HRs from the many meta-analyses mentioned in this paper, so as to elucidate the relative magnitudes of benefit or harm of many lifestyle factors (nutrition, cardiorespiratory fitness (CRF), grip strength, physical activity, obesity) with respect to cancer mortality. Table 1 also gives examples that depict the changes necessary to arrive at the associated cancer survival benefit. 

## 3. Specific Foods and Cancer Mortality

### 3.1. Red Meat

A frequently cited association between diet and cancer mortality is red meat. Pan et al. aggregated data from the Health Professionals Follow-up Study (HPFS) and the Nurses’ Health Study (NHS) to show a 19% increased risk of cancer mortality in those that ate roughly three servings (the highest quintile) of total red meat (which included both processed and unprocessed meat) vs. those that ate about half a serving (the lowest quintile) (HR = 1.19 [95% CI, 1.11–1.28]) [36]. The study also mentions a 10% increased risk in cancer death per serving of total red meat (HR = 1.10 [1.07–1.13]). For context, the standard serving size is classified differently based on the type of meat: one serving of unprocessed red meat is 85 g (a 3-ounce steak), while one serving of processed meat is classified as 13 g of bacon (one large/two small strips), 45 g of hot dogs (one frank), or 28 g of sausage, salami, bologna, and other processed red meats (one large deli slice) [36].

Other large epidemiological datasets within the United States have shown similar results. Sinha et al. examined over half a million people in the United States and demonstrated a roughly 20% increased risk of cancer mortality in the highest quintile of total red meat intake vs. the lowest in both men and women (HR = 1.22 [1.16–1.29] and HR = 1.20 [1.12–1.30], respectively) [39]. Combing both the Pan and Sinha studies, a meta-analysis by Wang et al. showed a 12% increased risk of cancer mortality per serving of total red meat (HR = 1.12 [1.10–1.14]) [12] (Table 1). It is worth mentioning that calorie intake was lower and physical activity was higher in the groups who ate the least red meat in the Pan and Sinha studies, both of which used multivariate analysis to correct for those factors. However, such group differences do suggest that additional differences between the quintiles might exist and for which complete accounting might be difficult.

Characterizing the risk of cancer related mortality imparted by red meat intake becomes more complicated when discussing processed vs. unprocessed red meat. When combing additional studies [21,40,41] to the Pan and Sinha papers, the Wang meta-analysis showed that intake of each additional serving of processed red meat per day intake increases cancer mortality risk by 8% (HR = 1.08 [1.06–1.11]). However, the associated per-serving increase was not dose-dependent and disappeared after an intake of about 35–40 g of processed meat, or roughly one serving ([12] Supplement). Looking at unprocessed meat, on the other hand, intake of an additional serving showed no association with cancer mortality (HR 1.06 [0.88–1.28]) [12], driven, in large part, by a pooled analysis of eight Asian cohorts (Cohorts from Bangladesh, China, Japan, and South Korea.) [42]. In the Asian cohorts, red meat actually appears to be protective from cancer mortality when comparing the third and first quintiles (HR 0.87 [0.78–0.96]). A large caveat in the Asian epidemiological data, however, is the fact that Asian countries have a much lower intake of red meat than the United States with a back-of-the-envelope estimate demonstrating that the highest quintile of red meat eaters in Asian countries consumes around half a serving of red meat per day, while the lowest quintile eats about 1/3rd of a serving [42].

Returning to the Pan study, the NNT comparing the highest and lowest quintiles of total red meat intake is 81 (raw, unadjusted calculation). This means that for every 81 people who decrease their red meat intake from three servings per day to less than one, over a 25-year period, one person would be spared death from cancer. In other words, the associated reduction in cancer mortality equates to abandoning two slices of bacon for breakfast and a 3-ounce steak for dinner, each day, for roughly two and a half decades.

### 3.2. Dietary Fiber

Increased dietary fiber intake appears to reduce one’s risk of cancer mortality. A large prospective cohort study performed in Europe showed an 18% reduction in cancer mortality when the highest quintile (≥28.5 g/d) was compared to the lowest quintile (<16.4 g/d), while correcting for several factors, including physical activity and calorie intake (adjustments for red meat intake did not change the associations and so were excluded from the final multivariate model), over a median follow-up of 12.7 years (HR = 0.82 [0.72–0.93] for men; HR = 0.82 [0.73–0.92] for women) [43]. Those in the highest quintile had a higher education level and were more physically active and appeared to eat more vegetables, larger amounts of red meat, and more calories compared to those in the lowest quintile of fiber intake. Fiber from cereals and vegetables, but not fruits, demonstrated reduced cancer mortality [43]. In another epidemiological study, this time based on a US cohort, Park et al. reported decreased cancer mortality in men (HR = 0.83 [0.76–0.92]) but not women (HR = 0.96 [0.85–1.08]) when the highest quintile of fiber intake was compared to the lowest [14]. There is some suggestion that the impact of fiber intake on cancer mortality might be dose-dependent with the greatest benefit conferred by increasing fiber consumption from low (< 13 g/day) to about 25 g of fiber per day [43]. This finding may explain why no statistical benefit was seen in women in the Park study as the highest quintile of fiber intake among women would have just met this threshold, with a median intake of 25.8 g per day.

It is worth noting that patients with the highest quintile of fiber intake in the Park et al. trial were more likely to have higher education, rate their health as very good/excellent, have a lower BMI, and be physically active, and were less likely to drink alcohol and consume red meat—all of which were corrected for using multivariate analysis. Although these characteristics were corrected for statistically, it is important to note the possibility of healthy-user bias in these studies as most baseline characteristics tend to favor the group already consuming the most fiber. Nevertheless, a pooled analysis of both of these studies demonstrated a 14% reduced risk in cancer death (HR = 0.86 [0.79–0.93]) when comparing those with high fiber intake to those with low fiber intake and a 6% reduced risk per 10-g per day increase of fiber intake (HR = 0.94 [0.91–0.97]) [13] (Table 1).

Using the data available from Park et al. [14], for every 49 people who currently eat less than 13 g of fiber per day, if they increase their daily fiber intake by 15–16 more grams—equivalent to five extra cups of strawberries, or an extra six cups of broccoli—one fewer cancer death would result over 9 years. This same group, should they increase their fiber intake by about 4 g per day—equivalent to one apple—one cancer death would be avoided per 77 people who adopt such a change.

### 3.3. Nuts

Using the NHS and HPFS, Bao at al. showed that nut consumption decreased cancer mortality by 11% in those that ate nuts five or more times per week vs. those that never ate nuts (HR = 0.89 [0.81–0.99]) [17]. Physical activity was higher in those that ate the most nuts. The authors mention that, based on the statistical assumptions, the benefit conferred by a confounder, such as physical activity, would have to be quite large to cancel out the signal for nut consumption’s reduction in cancer mortality. For instance, there would need to be a 30% difference between groups, with a confounding variable that conferred a 50% reduction in risk, to negate the effect of the nut consumption.

In another large epidemiological trial examining the participants of the Physicians Health Study, Hshieh et al. showed that consuming nuts five or more times per week, compared to less than one serving per week did not result in a significant reduction in cancer deaths, but showed only a trend in reducing cancer mortality (HR = 0.87 [0.66–1.15]) [16]. By combining the Hshieh et al. study with the data from Bao et al., a meta-analysis demonstrated that high versus low nut consumption yielded a 14% reduction in cancer mortality (HR = 0.86 [0.75–0.98]) [15] (Table 1).

When using the numbers found in the Hshieh paper to approximate the absolute benefit of nut intake at the epidemiological level, about 124 people would have to eat five servings (equivalent to a handful) or more of nuts—about 120 almonds—per week, over roughly 10 years, to prevent one cancer death.

### 3.4. Whole Grains

Whole grains have also been associated with decreased cancer mortality [44,45], possibly related to the fiber content of whole grains [44]. A meta-analysis by Chen et al. demonstrated an 18% benefit per 50 g of whole grain intake (HR = 0.82 [0.69–0.96]) [18] (Table 1). As context, in the NHS, the highest quintile of whole grain intake was roughly 36 g, and for the HPFS, it was around 53 g per day [37]. Although this analysis of the NHS and HPFS cohorts demonstrated an associated decrease in all-cause (HR = 0.91 [0.88–0.95]) and cardiovascular (HR = 0.85 [0.78–0.92]) mortality in the highest versus lowest quintiles of whole grain intake, no difference was seen with respect to cancer mortality (HR = 0.97 [0.91–1.04]) [37]. Additionally, the most favorable study—with a hazard ratio of 0.64 [0.56–0.72] per 50 g increase of daily whole grains—that was included in the Chen et al. meta-analysis only showed an associated 7% reduction in cancer mortality when comparing the fifth and first quintiles of per day whole grain intake (HR = 0.93 [0.88–0.99]) [44]. This is possibly due to the fact that even some of the people in the fifth quintile of whole grain intake do not report consuming greater than 50 g of whole grains per day [37,44]. To give that statement context, 50 g of whole grains is equivalent to about three-quarters of a cup of cooked quinoa or three cups of Cheerios (Table 1). (Note that quinoa is likely a healthier option given that it is a more complete whole grain. One hundred percent of the grains in quinoa are considered whole, compared to at least 50% in Cheerios (Table 1).)

Although whole grain intake displays a relatively modest HR between the highest and lowest quintiles, the NNT for whole grains is quite low, at 38 people. This may be explained by the very low age-adjusted hazard ratio (HR = 0.63 [0.59–0.67]) for highest versus lowest quintile of whole grain intake [37]. The difference in the age-adjusted HR, and the more rigorously, multivariate adjusted HR (HR = 0.97 [0.91–1.04]), highlights the healthy user bias in this population, and clarifies the limitations of using the NNT metric in epidemiology. Nonetheless, an NNT of 38 means that 38 people would have to eat roughly a half to two-thirds of a cup of oatmeal per day for roughly 25 years to prevent one cancer death.

### 3.5. Fruits and Vegetables

Maybe somewhat surprisingly, a large and widely-cited meta-analysis did not show reduced cancer mortality with higher rates of fruit and vegetable (HR = 0.97 [0.90–1.03]), fruit (HR = 0.99 [0.97–1.00]), or vegetable (HR = 0.99 [0.97–1.01]) consumption [19] (Table 1). A separate meta-analysis suggests that for every 200 g of fruit and vegetable consumption per day (roughly ten spears of asparagus, or one large bell pepper), there is an associated 3% decrease in total cancer (total cancer includes both cancer incidence and cancer mortality) (HR = 0.97 [0.95–0.99]) [46]. The magnitude is small and the paper does not comment on cancer mortality specifically. Overall, the results of this more recent meta-analysis are consistent with the first meta-analysis; the two studies support the idea that fruit and vegetable consumption are either not associated with improved cancer mortality or minimally so.

### 3.6. Other Foods

Before turning to exercise, it is important to mention that the relationship between cancer mortality and other food groups has also been studied and several different meta-analyses have been conducted (fish and poultry [20], dairy [22], eggs [25,47], legumes [23], and sugar-sweetened beverages (SSBs) [27,48]). No improvement in cancer mortality was seen between high versus low consumers of either fish (HR = 0.99 [0.94–1.05]) or poultry (HR = 0.96 [0.93–1.00]) [20], total dairy consumption (HR = 0.99 [0.92–1.07]) [22], or legumes (HR = 0.85 [0.72–1.01]) [23] (Table 1). Although a linear dose-response was not seen between intake of SSBs and cancer mortality [27,48], when looking at the highest versus the lowest consumers of SSBs (roughly two 12 ounce cans per day vs. <one can per month) there was an associated 6% increase in cancer related deaths (HR = 1.06 [1.01–1.12]) [27] (Table 1). As for eggs, when comparing the highest versus lowest egg intake categories, there was a 20% increased risk in cancer mortality (HR = 1.20 [1.04–1.39]; however, there was no increase in all-cause mortality, cardiovascular disease, coronary heart disease, stroke, or respiratory disease [25]. Importantly, the highest quintile of egg intake equals approximately half an egg per day [26]. In other words, the associated benefit from reducing egg intake stems from decreasing one’s omelet intake, roughly three eggs, from four times a month to once a month (Table 1).

### 3.7. A Grain of Salt

Lastly, some articles with respect to nutrition make very granular associations. These associations can be hard to interpret. For instance, every serving of whole milk per day increases prostate cancer mortality by 43% (HR = 1.43, [1.13–1.81]), but additional servings of total dairy (HR = 1.00 [0.97–1.04]), milk (HR = 1.02, [0.97, 1.08]), yogurt (HR = 0.6 [0.29–1.26]), cheese (HR 1.23 [0.76–1.99], butter (HR = 0.9 [0.45–1.80]), and skim/low fat milk (HR = 1.07 [0.95–1.20]) consumption do not [22]. At face value, the near 50% increase in prostate cancer mortality, per glass of whole milk, appears impressive; however, it is difficult to know what to do with this information. Should most men stop drinking whole milk? With a lack of association between other dairy products and no clear mechanism to explain this phenomenon—the mechanisms presented in the paper are not terribly convincing—it is truly difficult to draw an actionable conclusion in this instance.

These comments are not to minimize the importance of nutrition, but rather to highlight some of the limitations within nutritional epidemiology. By understanding such pitfalls, solitary examples, such as the one above, become less important and consistent patterns and repeated associations assume priority. With that said, the relative risks from foods that do showcase a more consistent association with cancer mortality (chose any of the previously mentioned foods) tend to be small. Whether the association with cancer mortality is detrimental (HRs in the vicinity of ~1.1–1.2) or favorable (HRs in the ~0.85–0.95 ballpark), the hazard ratio does not deviate far from 1, and that is when a risk or benefit exists. These ratios will be important to remember when turning to exercise, especially when considering cardiorespiratory fitness.

## 4. Exercise and Cancer Mortality

### 4.1. Cardiorespiratory Fitness

Cardiorespiratory fitness might have the largest impact on all-cause and cancer specific mortality compared to other lifestyle interventions. To focus on cancer mortality, Farrell et al. prospectively examined 38,419 men and split them into quintiles of cardiorespiratory fitness (CRF) as directly measured by treadmill testing at baseline [30]. In the case of CRF, the authors showed that the top quintile, compared to the worst quintile, had a 47% reduction in cancer mortality (HR = 0.53 [0.43–0.67]) when adjusted for age, examination year, smoking status, and chronic illness at baseline (Note that all the nutritional epidemiological studies correct for physical activity, a crude surrogate for CRF, whereas studies looking at CRF do not adjust for intake of certain foods.). Importantly, there was also a 29% reduced risk of cancer death when comparing the *second lowest* group of CRF to the lowest (HR = 0.71 [0.60–0.85]) [30]. These results emphasize the large benefit conferred even when CRF is only marginally improved. Expressed another way, the bottom quintile of CRF had an 89% and 41% increased risk of cancer death when compared to the top group or to the very next quintile, respectively. Compare these numbers to the highest vs. lowest quintiles (HR = 1.19 [1.11–1.28]) and the second vs. lowest quintiles (HR = 1.09 [1.02–1.16]) of red meat eaters [36].

To contextualize these results, taking 35 people from the lowest fitness group to the highest, over a median span of 17.2 years, would prevent one cancer death. Practically, this would mean a man who could sustain 6–7 min (A rough estimate for VO_2_ max.) of 12 min per mile pace (5 mph) would train to run 6 min per mile (10 mph) for those same 6–7 min (Table 1). Such a change in fitness is very difficult, and so examining the bottom and penultimate groups in CRF may be more instructive. The NNT does increase, but it rises to 63—still an impressive number. Furthermore, this equates to the same man who can sustain 6–7 min of 12 min-per-mile pace (5 mph) training to run just under 10 min per mile (6 mph) for that timespan (Table 2). With some training, a person can likely accomplish this feat within in a matter of months [49].

To complement the study evaluating CRF and cancer mortality in men, a similar study examined the association between CRF and cancer mortality in women. The authors prospectively evaluated 14,256 women over a median span of 15.2 years and compared cancer mortality among the first quintile of CRF (low CRF) with quintiles 2 and 3 combined (moderate CRF) and quintiles 4 and 5 combined (high CRF) [31]. They demonstrated a 32% reduced risk of cancer mortality when low CRF was compared to the high CRF group (HR = 0.68 [0.47–0.97]) and an 11% reduction in cancer mortality in the moderate CRF group (HR = 0.89 [0.67–1.18]). Although the confidence interval crosses one in the comparison between moderate and low CRF, the trend was statistically significant (*p*-trend = 0.03) [31]. To take the moderate CRF group versus the low CRF group in this trial, 61 women would have to improve their CRF from the bottom quintile to the second/third quintiles, to prevent one cancer death. Since women tend to have lower maximal oxygen consumption capacity than men on average [50], this translates to a woman who can sustain 6–7 min of roughly 13:20 min per mile (4.5 mph) pace training to maintain 11:30 min per mile (5.2 mph) pace for those same half dozen or so minutes (Table 3).

Additionally, both of these studies examined the relationship between BMI and body fat percentage, at baseline, with cancer mortality. These anthropometric variables showed a statistically significant trend for reduced mortality in men, but not women [30,31], although the *p*-value for a trend regarding BMI and cancer mortality in women was 0.08. Regardless, when accounting for specific variables, CRF conferred the largest and most consistent benefit related to risk of cancer death. Interestingly, fit, overweight men and women appeared to have similar cancer mortality risk when compared to unfit, normal weight individuals [30,31]. Additionally, other results suggest that, when prospectively following only overweight and obese men, the direction and magnitude of better fitness on the risk of dying from cancer remains [51]. In fact, improved CRF in those with excess adiposity is possibly more protective against the associated risk of cancer mortality than improved CRF in those with normal weight. For instance, highly fit (>10 METs), overweight men compared to minimally fit (<5 METs) overweight men had a 79% reduced risk of cancer mortality (HR = 0.21 [0.14–0.34]). Even moderately fit (5–10 METs) overweight men, compared to minimally fit overweight men, demonstrated a large associated benefit on cancer mortality (HR = 0.52 [0.38–0.69]) [51]. The hazard ratios are even (slightly) better for obese men (HR = 0.17 [0.08–0.34] for high vs. low CRF; HR = 0.45 [0.31–0.66] moderate vs. low CRF) [51]. To emphasize this point, moderately fit, overweight and obese men share a similar HR to that of highly fit, normal weight men, when these groups are compared to their respective least fit peers.

Other studies consistently show the benefit of CRF on cancer mortality [52,53,54,55]. Pooling many of these results together, a systematic review and meta-analysis demonstrated that the highest versus the lowest levels of CRF yielded a 45% reduction in cancer mortality (HR = 0.55 [0.47–0.65]) with a 20% reduction extending to intermediate versus low CRF levels (HR = 0.80 [0.67–0.97]) [29] (Table 1). The Farrell et al. studies [30,31] are consistent with this HR. Additionally, the results from the meta-analysis hold for high vs. low fitness when adjusting for adiposity (HR = 0.55, 95% CI [0.46–0.66]) [29], suggesting that CRF outweighs the effect of obesity, which itself is associated with worse cancer mortality [34,38].

These results highlight that even moving from a low to a moderate level of CRF has an associated magnitude of benefit on cancer mortality that compares more favorably to either low red meat, high fiber, high nut, or high whole grain consumption [12,13,15,37] (Table 1).

### 4.2. Other Metrics of Physical Health: Strength and Physical Activity

Besides cardiorespiratory fitness, there are several other ways to evaluate physical health, such as strength and physical activity, both of which display reductions in cancer mortality. For instance, a meta-analysis by López-Bueno showed that the bottom compared to the top third in grip strength—a proxy for overall muscle strength—was associated with a 27% increased risk of cancer mortality (HR = 1.27 [1.01–1.59]) [32] (Table 1). Even the lowest compared to the middle group of grip strength had a 12% increased risk of cancer-related death (HR = 1.12 [1.03–1.23] [32] (Table 1). In a similar vein, a separate meta-analysis demonstrated that the most highly active individuals had a 17% reduction in the risk of dying from cancer (HR = 0.83 [0.79–0.87]) [33] (Table 1). Similar to CRF, a large portion of the benefit in physical activity came from a small improvement in the lowest activity category. This is highlighted by the fact that engaging in five MET hours per week—the equivalent of taking a leisurely, one-hour walk two times per week—compared to few or no MET hours per week, yielded a 12% reduction in cancer mortality (HR = 0.88 [0.82–0.95]) [33] (Table 1). Like CRF, the examples of strength and activity level showcase the benefits of exercise on cancer mortality.

## 5. Obesity

Intertwined with the relationship between nutrition and CRF/strength/physical activity and cancer mortality is adiposity. Obesity, for a long time, has been associated with increased cancer mortality. A landmark study, published in the New England Journal of Medicine in 2003, showed that non-smokers with a BMI of 25–29.9, compared to normal weight individuals (BMI 18.5–24.9), had an associated 11% and 14% increased risk of cancer mortality in men and women, respectively (HR = 1.11 [1.05–1.18] for men; HR = 1.14 [1.09–1.18] for women) [38]. This risk increased to 38% and 33% in non-smokers with a BMI of 30–34.9 for men and women, respectively (HR = 1.38 [1.24–1.52] for men; HR = 1.33 [1.25–1.41]) for women) [38]. Additionally, a recent meta-analysis, published in JAMA in 2021, showed that obesity (BMI ≥ 30) resulted in a 17% increase in cancer specific mortality compared to non-obese (BMI < 30) people (HR = 1.17 [1.12–1.23]) [34] (Table 1). In addition, several other meta-analyses have shown that obesity is associated with cancer mortality in a wide range of disease sites, such as prostate, breast, pancreas, and liver cancers [56,57,58,59], with many of these studies demonstrating a consistent dose response per increase in BMI [56,57,58,59,60]. Using the NEJM paper as an example, for roughly every 62 people who decrease their BMI from ≥30 to ≤24.9, and for every 130 people who decrease their BMI from 25–29.9 to ≤24.9, one cancer death would be prevented. For reference, a BMI change from obese or overweight to the normal range is the equivalent of a five foot four woman who weighs 190 pounds (BMI 32.6), or a woman of similar stature who weighs 160 pounds (BMI 27.5), losing 50 or 20 pounds, respectively (Note: a 5′ 4” woman who weighs 140 pounds has a BMI of 24.0).

Obesity and nutrition, in particular, are interconnected through the idea that certain foods, such as highly palatable, processed foods and/or sugars and refined carbohydrates, contribute to overeating and subsequent weight gain [61,62,63]. This means that some foods might exert their association with cancer mortality via worsening body composition, and not through the nutrient per se. For instance, a diet that includes SSBs might contribute to the overconsumption of calories, thereby causing weight gain, which in turn leads to elevated cancer mortality. Population based studies, however, do correct for BMI and calorie consumption in an attempt to minimize this type of effect. Although calorie intake—outside of its contribution to adiposity—was not further explored in this review, it is still an important component to mention, especially given the complex relationship that foods might have on calorie consumption [61]. All this fails to mention the aging-related benefits [64] and possible anti-cancer properties [65] of overeating’s converse, fasting or calorie restriction. At the end of the day, we are left with nutrition, and/or exercise, and/or weight management (and/or medications—beyond the scope of this paper) to reduce the associated risk of cancer mortality. So, ideally, when it comes to lifestyle intervention, all three of these modifications are attempted—diet, exercise, and weight control—in the hopes of reducing cancer mortality. However, if the clinician or patient should choose one behavioral modification, it should be exercise.

## 6. After Thoughts: Lifestyle Interventions after a Diagnosis of Cancer

This review did not deeply examine the benefits of nutrition or physical activity or CRF after a cancer diagnosis. However, in short, there is significant epidemiological evidence that physical activity improves survival outcomes in this space [66,67,68,69,70,71]. The magnitude of benefit is impressive, even when comparing the moderately physically active to the not-at-all physically active with HRs on the scale of 0.5–0.8. These results also hold in a recent prospective cohort study, nested within a randomized trial, that showed light to moderate exercise for ≥1.5 h per week after initial diagnosis of stage III colon cancer resulted in a 21.4% absolute difference in disease free survival (87.1% vs. 65.7%, *p* < 0.001) [72]. This corresponds to an impressive NNT of less than five to prevent one event. The benefit also applies to the more concrete measure of CRF, not just for physical activity. For instance, in 1632 cancer patients who had exercise treadmill testing, moderate CRF (the ability to run 6–7 min of 12-min-per-mile pace) compared to low CRF (capability of sustaining 6–7 min of 13+ minutes-per-mile pace) showed an improved all-cause (HR = 0.38 [0.28–0.52]) and cancer-specific mortality (HR = 0.40 [0.26–0.60]) [73]. One caveat is that the median time to CRF testing was 7 years from diagnosis, which introduces a significant selection bias. The hazard ratios are, nonetheless, impressive.

Nutrition after a diagnosis of cancer also likely plays a role in outcomes. Several meta-analyses suggest that post-diagnosis diets, such as a Western diet (red meats, processed meats, refined grains, sweets, and desserts) and a prudent diet (fruit and vegetables, whole grains, poultry, and low-fat dairy products) are associated with worse or improved overall survival, respectively [74,75]. A meta-analysis by Schwedhelm et al. highlighted that the highest vs. lowest adherence to a Western diet, post-diagnosis, had an associated 51% increased risk of all-cause mortality (HR = 1.51 [1.24–1.85]), whereas the highest compared to the lowest adherers to a prudent diet, post-diagnosis, had an associated 33% decreased risk of overall mortality (HR = 0.77 [0.60–0.99]) [75]. However, individual foods—fruit, vegetable, dairy, meat, fish, bread, or egg consumption—were not associated with either better or worse mortality when consumed post-diagnosis [75]. Additionally, no dietary patterns or foods were associated with cancer recurrence, suggesting the survival benefit might be unrelated to decreased cancer deaths (cancer mortality, unfortunately, was not assessed) [75]. Lastly, a prospective cohort trial of patients participating in the CALGB 89803 adjuvant therapy trial for stage III colon cancer by Fuchs et al. demonstrated an associated 67% worse disease free survival in those who drank ≥ 2 SSBs per day compared to < 2/month, after a median follow-up of 7.3 years (HR = 1.67 [1.04–2.68]) [76]. However, there was no associated improvement in overall survival (HR = 1.51 [0.87–2.63]). The magnitude of recurrence risk with high SSB consumption is quite high in the Fuchs et al. paper (HR = 1.67) and might suggest that post-diagnosis diet, at least when it comes to SSBs and colorectal cancer, might be more important than pre-diagnosis diet. Such findings may be explained on the molecular level, as high-fructose corn syrup—a main ingredient in SSBs—has been shown to increase intestinal tumor growth in mice [77].

## 7. Discussion

Several guidelines exist for cancer patients and cancer survivors regarding diet and physical activity [1,78,79,80]. However, outside broad recommendations [81], there are few concrete guidelines concerning lifestyle changes that, if taken prior to a diagnosis of cancer, might reduce cancer deaths. This lack of specificity is understandable, given the difficulty in interpreting large, population-based data at the individual level. Unfortunately, this reality leaves the patient and clinician with the all-too common and hackneyed advice of “eat healthy” and “exercise more.” When we do try and interpret the data, we must make sense of relative risks and hazard ratios. As an example, taking the high versus low quintiles of fiber intake [13], many realize that a hazard ratio of 0.86 represents an associated 14% reduction in cancer mortality risk. However, the interpretation often stops there. “Eat more fiber” becomes the prevailing, and rather unhelpful, talking point. How does one take advantage of that 14% relative risk reduction? Does everyone receive the benefit? What are the actual changes required? Over what time period?

This article was an attempt to field some of these questions. By taking the available epidemiological data and converting the percentages and hazard ratios found at the population level into easier-to-digest terms and vignettes (Table 1, Table 2 and Table 3), we aimed to provide rough suggestions on how an individual might change his or her behavior to potentially (the word ‘potentially’ is important here because epidemiological studies establish association, not causation) derive a cancer mortality benefit. We did this through the NNT, which is a very rough metric that suggests the number of individuals who must make a behavioral change in order for one person to benefit. To get a sense of how this NNT might change across quintiles, we included comparisons of the lowest quintile with both the second lowest and highest quintiles of men (Table 2) and women (Table 3).

The numerous NNTs calculated in this study were not adjusted for confounders and were derived from raw data. So, the NNT serves as a very rough approximation. If we had access to the data required to calculate an adjusted NNT, we would expect the NNT to be higher (less favorable) than what we report in Table 2 and Table 3. This point is especially salient with regards to the nutritional data, as those cohort studies must adjust for a larger number of confounders in the multivariate analyses compared to the CRF, strength, and physical activity cohorts. A limitation of the NNT metric is its reliance on raw data, which becomes most apparent when looking at the rather low NNT for whole grains; the NNT of 34–41 is likely overly generous (Table 1 and Table 2). This low NNT is explained by the low age-only adjusted hazard ratio (HR = 0.63 [0.59–0.67]), compared to the more rigorously, multivariate-adjusted one (HR = 0.97 [0.91–1.04]) [37]. Regardless, we feel that the NNT, with its subsequent conversion into clear and concrete changes (Table 2 and Table 3), might be a valuable resource for both patients and clinicians.

The largest benefit regarding cancer mortality for the least amount of change appears to be when a person moves from the lowest level of CRF to the second lowest quintile. This conclusion is supported by the modest change in VO_2_ max required to move a person from the lowest to the penultimate CRF quintile (i.e., training a person to run ~10:30 mile-pace for 6–7 min from their current baseline of ~12:30 mile-pace), coupled with the second lowest overall hazard ratio (Table 1). (Note: the best HR is found when comparing the highest vs. lowest levels of CRF (Table 1).) In other words, moving from the lowest to the next lowest group in CRF provides a greater associated benefit regarding cancer mortality than moving from the worst to the best quintiles of red meat, fiber, nut, and whole grain intake (Table 1, Table 2 and Table 3).

Looking at other measures of fitness, such as strength and physical activity, we see hazard ratios that are on par with, if not slightly better than, the best hazard ratios from the nutritional studies (Table 1). Although the hazard ratios for grip strength and physical activity are not quite as pronounced as those seen for CRF (Table 1), the large improvement, especially when moving from the worst to the second worst group, is still noticeable (Table 1). When juxtaposing this idea with the nutritional data, a relatively small change in strength or physical activity yields a similar impact on cancer mortality compared to larger dietary changes. For example, taking a leisurely, one-hour walk two times per week, in relation to inactivity, and increasing one’s nut intake from zero to 10 walnuts per day, are associated with similar reductions in cancer mortality [12,33] (Table 1). We feel that asking someone to take two long, or four short, walks per week, is a smaller ask than recommending that a person increase their nut consumption by five servings each day.

Lastly, it is hard not to notice that the meta-analyses examining CRF (high vs. low and moderate vs. low), grip strength (high vs. low), and physical activity (high vs. low) with cancer mortality demonstrate the first, second, third, and fifth most favorable hazard ratios found in the 16 evaluated meta-analyses (Table 1).

### Limitations

When it comes to epidemiological data, the NNT is not a scientifically rigorous term. We feel this limitation is outweighed by the rough contextualization this number provides. Another criticism might be the fact that only one meta-analysis is reported for each lifestyle modification in Table 1. This is a fair critique; however, we feel that the choice of an individual meta-analysis was systematic and consistent with both PRISMA and/or MOOSE criteria. When results did return multiple meta-analyses on a similar intervention, the hazard ratios were remarkably similar. Take red meat [12,82], dietary fiber [13,83], nut [15,84], and egg [25,47] consumption, as well as CRF [29,85], as proof of this statement. For this reason, the conclusions one might draw from Table 1 would be similar even if a different, maybe less well-vetted, meta-analysis was chosen. Finally, out of convenience, we used relative risk and hazard ratio synonymously. If a study or meta-analysis used the term ‘relative risk’, it was reported herein as a hazard ratio. Although differences do exist between those two terms, we feel that the similar follow-up time and similar prospective cohort study designs make equating relative risk with hazard ratio a reasonable and practical consideration.

## 8. Summary

In a study that videotaped one-hundred real-life consultations between patients and primary care providers, lifestyle recommendations were mentioned in 86% of consults [86]. Of these, the lifestyle advice that was given concerned weight one-quarter of the time, diet two-thirds of the time, and physical activity one-third of the time [86]. Based on the hazard ratios reported herein, however, much more emphasis should be placed on physical activity, and more specifically on improving CRF. Although how to motivate people to exercise is complicated and out of the scope of this review, recommending exercise—encouraging any type of physical activity (walking, golfing, weight lifting, gardening, etc.)—is likely easier, simpler, and more beneficial than dietary advice. This is not to minimize the importance of optimal nutrition; rather, this piece presents a way to contextualize the different magnitudes of association that certain foods or baseline CRF have with cancer mortality. The reader can use this information to make a more informed decision about the relative benefits and realistic implementation of each lifestyle modification described in this paper. It is difficult for the authors, though, to not end by emphasizing the large association of high cardiorespiratory fitness—and to an extent even moderately improved CRF—with a reduced risk of dying from cancer.

## Figures and Tables

**Table 1:** Aggregation of the many adjusted hazard ratios from the meta-analyses cited in this paper. Examples are given to suggest the changes required to potentially derive the associated cancer mortality benefit.

Lifestyle Factor Associated with Cancer Mortality	Hazard Ratio (95% CI)	Hazard Ratio Relative to _______	Examples of Lifestyle Intervention to Derive Associated Cancer Mortality Benefit
Unprocessed Red Meat [12]	HR = 1.03 (0.95–1.13)	per daily serving	Reduction by: 1 large strip of bacon (~13 g/slice); 1 hot dog (45 g/frank); 2 slices of salami, bologna (~14 g/slice), per day.
Processed Red Meat [12]	HR = 1.08 (1.06–1.11) *	per daily serving	One fewer 3-ounce steak (~85 g) per day.
Total Red Meat [12]	HR = 1.12 (1.10–1.14)	per daily serving	One fewer of any combination of the examples given in the processed and unprocessed red meats sections per day.
Fiber [13]	HR = 0.86 (0.79–0.93)	High vs. low (~25+ g/day vs. ~10 g/day [14])	A daily meal plan of: a cup of oatmeal (5 g fiber) topped with a half cup of raspberries (4 g fiber) for breakfast plus an orange (3 g fiber) and a large handful (20 nuts) of almonds (3 g fiber) for lunch plus one cup of chopped broccoli (5 g fiber) over a cup of quinoa (5 g fiber) for dinner.
Fiber [13]	HR = 0.94 (0.91–0.97)	per 10 g/day	An additional: 1 cup of canned baked beans; 2½ cups of Brussel sprouts; 3 large bananas; 5 slices of whole wheat bread, per day.
Nuts [15]	HR = 0.86 (0.75–0.98)	High vs. low (Roughly > 5 servings per week vs. roughly < 1 serving per month/never [16,17])	An additional: 115 almonds, 90 cashews, 70 walnuts, 95 pecans, 245 pistachios per week.
Whole Grains [18]	HR = 0.82 (0.69–0.96)	per 50 g/day	An additional **: ⅔ cups of old-fashioned oats †; ¾ cup cooked quinoa (¼ uncooked) †; 3 slices of 100% whole wheat bread ‡; 3 cups of Cheerios ‡, per day.
Vegetables [19]	HR = 0.99 (0.97–1.01)	per daily serving	N/A
Fruit [19]	HR = 0.99 (0.97–1.00)	per daily serving	N/A
Fish [20]	HR = 0.99 (0.94–1.05)	High vs. low (roughly 3×/week vs. <1×/month [21])	N/A
Poultry [20]	HR = 0.96 (0.93–1.00)	High vs. low (roughly 2×/week vs. <1×/month [21])	N/A
Total Dairy [22]	HR = 0.99 (0.92–1.07)	High vs. low (roughly ≥2×/day vs. <0.5/day)	N/A
Legumes [23]	HR = 0.85 (0.72–1.01)	High vs. low (roughly 27.8 g/day vs. 0 g/day [24])	N/A
Eggs [25]	HR = 1.20 (1.04–1.39)	High vs. low (roughly half an egg/day vs. ≤3 egg/month [26])	Decreasing egg consumption from 12 medium-sized eggs, or 4 omelets, per month to about 3 medium-sized eggs, or one omelet, per month.
SSBs ◊ [27]	HR = 1.06 (1.01–1.12)	High vs. low (≥2 SSBs/day vs. <1 SSB/month [28])	Decreasing consumption of two 12-ounce cans of soda (~80 g of sugar) per day to less than one 12-ounce can per month.
CRF [29]	HR = 0.55 (0.47–0.65)	High vs. low (~13 METs vs. ~8.5 METs for men; ~12 METs vs. ~7 METs for women [30,31])	Training a man who can sustain 6–7 min ^®^ of ~12 min per mile pace (5 mph) to sustain 6–7 min of ~6-min per mile (10 mph); Training a woman who can sustain 6–7 min of ~13:20 min per mile pace (4.5 mph) to sustain 6–7 min of ~7:15-min per mile (8.3 mph).
CRF [29]	HR = 0.80 (0.67–0.97)	Moderate vs. low (~11 METs vs. ~8.5 METs for men; ~9 METs vs. ~7 METs for women [30,31])	Training a man who can sustain 6–7 min of ~12 min per mile pace (5 mph) to sustain 6–7 min of ~10-min per mile (6 mph); Training a woman who can sustain 6–7 min of ~13:20 min per mile pace (4.5 mph) to sustain 6–7 min of ~11:30-min per mile (5.2 mph).
Hand grip [32]	HR = 1.27 (1.01–1.59)	Lowest third vs. highest third	Grip strength of roughly < 20 kg vs. >30 kg; this is the force exerted on a hand dynamometer.
Hand grip [32]	HR = 1.12 (1.03–1.23)	Lowest third vs. middle third	Grip strength of roughly < 20 kg vs. roughly 20–30 kg; this is the force exerted on a hand dynamometer.
Physical Activity € [33]	HR = 0.83 (0.79–0.87)	High vs. low (very roughly ≥ 25 MET-hours per week vs. little to no MET-hours per week) ¥	~1 h per day (7 h over a week) of walking at a moderate pace (3 mph); running at 10 min-per-mile pace for 30 min 5x/week; playing 2 rounds of golf per week (using a golf cart); 4 h per week of resistance training (lifting weights) plus 2 h per week of gardening plus 1 h of playing tennis.
Physical Activity € [33]	HR = 0.88 (0.82–0.95)	5 MET-hours per week vs. little to no MET-hours per week ¥	~1 h of a leisurely bike ride per week; gardening about two hours per week; ~2 h per week of casual walking.
Obesity [34]	HR = 1.17 (1.12–1.23)	Obese (BMI ≥ 30) vs. non-obese (BMI < 30)	A 5′ 9” man, weighing 220 pounds (BMI 32.5), who loses 25 pounds (BMI 28.8). A 5′ 4” female, weighing 190 pounds (BMI 32.6), who loses 20 pounds (BMI 29.2).

* The associated per serving increase was not dose-dependent and disappeared after about an intake of 35–40 g of processed meat, or roughly 1 serving ([12] Supplement). ** Whole grain content can be difficult to calculate. The whole grain stamp (from the whole grain council) was used to estimate whole grain content of foods (https://wholegrainscouncil.org/find-whole-grains/stamped-products, accessed on 2 February 2023). † 100% whole grain stamp: all the grains in the food come from whole grains. ‡ 50% whole grain stamp: at least half of the grains in the food come from whole grains (many, but not all, 100% whole wheat breads have the 50% whole grain stamp). ◊ SSB serving size is most commonly defined as one standard glass, bottle, or can. SSBs are defined as caffeinated colas, caffeine-free colas, noncola carbonated sodas, and noncarbonated sugar-sweetened beverages, such as fruit punches, lemonades, or other fruit drinks. Note: fruit juice is not included as an SSB (although it is unclear if these drinks should also be included in the SSB label [35]). ^®^ Roughly the length a person can sustain their VO_2_ max. € Physical activity is more often measured via subjective report, in comparison to CRF or grip strength, which are directly measured. ¥ One MET hour = METs of an activity × duration of the activity (in hours). For examples of activities and their associated METs, use the 2011 Compendium of Physical Activities, easily accessible online.

**Table 2:** Adjusted hazard ratios and calculated NNTs for several different lifestyle factors and their association with cancer mortality in men. The NNTs are based on raw data and so are uncorrected for confounders. The study used to calculate the respective NNT was chosen if it had a similar hazard ratio (HR) to the one found in the chosen meta-analysis on the particular subject. The HR, which gives the adjusted relative risk for cancer mortality, accompanies the NNTs for a more accurate, but sometimes conceptually more challenging, description of the data.

Epidemiological Factor	N	Follow-Up	Age	Adjusted Hazard Ratio (Second Quintile Compared to Lowest)	Raw NNT * (Second Quintile Compared to Lowest)	Interpretation of NNT	Adjusted Hazard Ratio (Highest Quintile Compared to Lowest)	Raw NNT * (Highest Quintile Compared to Lowest)	Interpretation of NNT
Total Red meat [36]	37,698	Up to 22 years	40–75 (range)	1.05 (0.94–1.18) †	110 in favor of 0.62 servings per day vs. 0.22 servings per day	110 men would have to eat 1 more small slice of bacon per day, over roughly 2 decades, to avoid one cancer death.	1.24 (1.09–1.40) †	73 in favor of 0.22 servings per day vs. 2.36 per day	73 men would have to avoid 2 small slices of bacon for breakfast and one 3-ounce steak for diner per day, over roughly two decades, to avoid one cancer death.
Fiber [14]	219,123	9 years (mean)	50–71 (62, mean)	0.98 (0.91–1.04) †	94 in favor of 16.4 g/day vs. 12.6 g/day	94 men would need to increase their fiber intake by one medium sized apple per day, over 9 years, to avoid one cancer death.	0.83 (0.76–0.92) †	42 in favor of 29.4 g/day vs. 12.6 g/day	42 men would have to increase their fiber intake by roughly 15 g (1 cup of lentils or 6 cups of broccoli) per day, over 9 years, to prevent one cancer death.
Nuts [16]	20,742	9.6 years (mean)	66.6 (mean)	0.91 (0.77–1.08) ‡	120 in favor of 1–3 servings per week vs. <1 serving per week	120 men would need to increase their nut consumption by ~20 walnuts per day, over 10 years, to avoid one cancer death.	0.87 (0.66–1.15) ‡	124 in favor of ≥5 servings per week versus <1 serving per week	124 men would need to increase their nut consumption by ~70 walnuts per week, over 10 years, to avoid one cancer death.
Whole Grains [37]	51,529	Up to 24 years	53.2 (mean)	1.01 (0.92–1.11) ‡	46 ◊ in favor of ~14 g/day vs. 5.8 g/day	46 men would have to consume two-thirds of a cup of Cheerios per day over 24 years to avoid one cancer death.	0.95 (0.86–1.05) ‡	34 ◊ in favor of 52.6 g/day vs. 5.8 g/day	34 men would have to consume a little more than half a cup of oatmeal per day over 24 years to avoid one cancer death.
CRF [30]	38,410	17.2 years (mean)	43.8 (mean)	0.71 (0.60–0.85) †	63 in favor of 10.2 maximal METs vs. 8.4 maximal METs	If 63 men, who can currently run 12-min-per-mile pace for 6–7 consecutive minutes, improve their fitness as to be able to sustain 10-min-per-mile pace for the same 6–7 min, one cancer death will be prevented over 17 years.	0.53 (0.43–0.67) †	35 in favor of 14.9 maximal METs vs. 8.4 maximal METs	If 35 men, who can currently run 12-min-per-mile pace for 6–7 consecutive minutes, improve their fitness as to be able to sustain 6-min-per-mile pace for the same 6–7 min, one cancer death will be prevented over 17 years.
Obesity [38]	107,030	16 years	57 (mean)	1.11 (1.05–1.18) ¥	144 in favor of normal weight (BMI 18.5–24.9) vs. overweight (BMI 25.0–29.9)	If 144 men who are 5′ 9” and weigh 190 pounds lose 30 pounds, one cancer death will be prevented over 16 years (note: a 160-pound man at the same height has a BMI of 23.6).	1.38 (1.24–1.52) ¥	45 in favor of normal weight (BMI 18.5–24.9) vs. obese (BMI 30.0–34.9)	If 45 men who are 5′ 9” and weigh 220 pounds (BMI 32.5) lose 60 pounds, one cancer death will be prevented over 16 years (note: a 160-pound man at the same height has a BMI of 23.6).

* Raw NNT is an uncorrected value and is derived from the raw data from individual papers. The fully adjusted hazard ratios are provided for more context. Statistical significance for the trend in the individual paper is also noted. The studies without statistically significant results were included since information from meta-analyses shows that the specific lifestyle change has a statistically significant association with cancer mortality once additional studies are added. † *p*-trend < 0.001; ¥ *p*-trend < 0.05; ‡ *p*-trend > 0.05; ◊ The age-only adjusted hazard ratio for whole grains was quite low comparing both Q5 (HR = 0.67 [0.61–0.74]) and Q2 (HR = 0.84 [0.76–0.92]) to Q1, suggesting a higher level of confounding in comparison to some of the other studies. This example highlights a limitation of the raw NNT.

**Table 3:** Adjusted hazard ratios and calculated NNTs for several different lifestyle factors and their association with cancer mortality in women. The NNTs are based on raw data and so are uncorrected for confounders. The study used to calculate the respective NNT was chosen if it had a similar hazard ratio (HR) to the one found in the chosen meta-analysis on the particular subject. The HR, which gives the adjusted relative risk for cancer mortality, accompanies the NNTs for a more accurate, but sometimes conceptually more challenging, description of the data.

Epidemiological Factor	N	Follow-Up	Age	Adjusted Hazard Ratio (Second Quintile Compared to Lowest)	Raw NNT * (Second Quintile Compared to Lowest)	Interpretation of NNT	Adjusted Hazard Ratio (Highest Quintile Compared to Lowest)	Raw NNT * (Highest Quintile Compared to Lowest)	Interpretation of NNT
Total Red meat [36]	83,644	Up to 28 years	34–59 (range)	1.05 (0.97–1.14) †	132 in favor of 1.04 servings per day vs. 0.53 per day	132 women would have to eat 1 more slice of bacon per day, over roughly 2 decades, to avoid one cancer death.	1.17 (1.08–1.24) †	85 in favor of 0.53 servings per day vs. 3.10 per day	85 women would have to avoid 2 pieces of salami for lunch and one 3-ounce steak for diner, per day, over roughly two decades, to avoid one cancer death.
Fiber [14]	168,999	9 years (mean)	50–71 (62, mean)	0.93 (0.85–1.01) ‡	69 in favor of 14.3 g/day vs. 10.8 g/day	69 women would need to increase their fiber intake by one medium sized apple per day, over 9 years, to avoid one cancer death.	0.96 (0.85–1.08) ‡	63 in favor of 25.8 g/day vs. 10.8 g/day	63 women would have to increase their fiber intake by roughly 15 g (1 cup of lentil or 6 cups of broccoli) per day, over 9 years, to prevent one cancer death.
Whole grains [37]	121,700	Up to 26 years	50.2 (mean)	1.02 (0.94–1.10) ‡	53 ◊ in favor of ~10 g/day vs. 4.3 g/day	53 women would have to consume roughly two-thirds of a cup of Cheerios per day for 26 years to prevent one cancer death.	0.99 (0.91–1.07) ‡	41 in favor of 35.6 g/day vs. 4.3 g/day	41 women would have to consume a little over one third of a cup of oatmeal per day over 26 years to prevent one cancer death.
CRF [31]	14,256	15.2 years (mean)	43.8 (mean)	0.89 (0.67–1.18) ¥	61 in favor of moderate vs. low CRF (8.9 METs vs. 7.0)	If 61 women, who can currently run 12:30-min-per-mile pace for 6–7 consecutive minutes, improve their fitness as to be able to sustain 11:30-min-per-mile pace for the same 6–7 min, one cancer death will be prevented over 15 years.	0.68 (0.47–0.97) ¥	40 in favor of high vs. low CRF (11.4 METs vs. 7.0)	If 40 women, who can currently run 12:30-min-per-mile pace for 6–7 consecutive minutes, improve their fitness as to be able to sustain 8:20-min-per-mile pace for the same 6–7 min, one cancer death will be prevented over 15 years.
Obesity [38]	276,564	16 years	57 (mean)	1.14 (1.09–1.18) †	170 in favor of normal weight (BMI 18.5–24.9) vs. overweight (BMI 25.0–29.9)	If 170 women who are 5′ 4” and weigh 160 pounds (BMI 37.5) lose 25 pounds, one cancer death will be prevented over 16 years (note: a 140-pound woman at the same height has a BMI of 24.0).	1.33 (1.25–1.41) †	70 in favor of normal weight (BMI 18.5–24.9) vs. obese (BMI 30.0–34.9)	If 70 women who are 5′ 4” and weigh 190 pounds (BMI 37.5) lose 50 pounds, one cancer death will be prevented over 16 years (note: a 140-pound woman at the same height has a BMI of 24.0).

* Raw NNT is an uncorrected value and is derived from the raw data from individual papers. The fully adjusted hazard ratios are provided for more context. Statistical significance for the trend in the individual paper is also noted. The studies without statistically significant results were included since there is information from meta-analyses that the lifestyle change has a statistically significant association with cancer mortality once additional studies are added. † *p*-trend < 0.001; ¥ *p*-trend < 0.05; ‡ *p*-trend > 0.05; ◊ Similar to the case with men, the age-only adjusted hazard ratio for whole grains in women was quite low comparing both Q5 (HR = 0.60 [0.56–0.65]) and Q2 (HR = 0.80 [0.74–0.86]) to Q1, again suggesting a higher level of confounding in comparison to some of the other studies. This example highlights a limitation of the raw NNT.

## Data Availability

For any inquiries regarding the calculations of the NNTs, or other questions concerning the information reported in this article, please reach out to Timothy P. Dougherty at timothy.dougherty@fccc.edu.
